# Impact of the COVID-19 Shutdown on Mental Health in Appalachia by Working Status

**DOI:** 10.13023/jah.0301.03

**Published:** 2021-01-24

**Authors:** Erin N. Haynes, Timothy J. Hilbert, Susan C. Westneat, Kate Leger, Katie Keynton, Heather M. Bush

**Affiliations:** University of Kentucky, Lexington, Kentucky, erin.haynes@uky.edu; University of Cincinnati, hilbertj@ucmail.uc.edu; University of Kentucky, swest1@email.uky.edu; University of Kentucky, kate.leger@uky.edu; University of Kentucky, katie.keynton@uky.edu

**Keywords:** Appalachia, mental health, COVID-19, essential workers, financial impact

## Abstract

**Introduction:**

To slow the spread of COVID-19 in the United States, businesses shutdown in Spring 2020. Research has indicated the impact on frontline workers, yet little is known about the impact on those who were not working outside the home or switched to working remotely.

**Purpose:**

The purpose of this report is to identify the financial and healthcare issues and mental health impact of the COVID-19 shutdown on Appalachians by worker categories.

**Methods:**

An online survey was administered from May 8 – June 6, 2020 to a convenience sample of previous research participants and shared through social media networks, i.e., snowball sampling. Questions investigated mental health, financial and healthcare issues, and employment status. Survey responses were summarized by percentages overall and by working categories.

**Results:**

Of the 751 participants, 276 (42%) resided in Appalachia. After removing 17 who lost their job due to COVID-19, 59 (23%) indicated not working outside the home prior to COVID-19, whereas 111 (43%) and 89 (34%) switched to working remotely and continued working outside the home during the shutdown. Respondents were predominately Caucasian and female. Nearly a quarter of participants had lost or reduced income due to the shutdown. Approximately half missed or postponed healthcare appointments. Mental health impacts were similar across the groups, with half of the respondents reporting anxiety due to COVID-19.

**Implications:**

Further research is needed to explore health outcomes associated with missed healthcare appointments during the shutdown. Mental health support may be needed equally by those isolated at home and frontline workers.

## INTRODUCTION

In Spring of 2020 the Severe Acute Respiratory Syndrome Coronavirus 2 (SARS-CoV-2) and Coronavirus Disease 2019 (COVID-19) was spreading rapidly in the U.S. To slow the spread, schools and businesses were asked to shut down. Essential or frontline workers such as healthcare providers, social workers, grocery store employees, delivery drivers, gas station employees, and others continued working outside the home. Non-essential businesses faced an unprecedented economic disruption and struggled with maintaining employment for their employees. In a survey of 5,800 small businesses, Bartik and colleagues^1^ found that 43% of businesses had temporarily closed primarily due to reductions in demand and employee health concerns as a result of COVID-19.

The pandemic has taken an immense toll on rural areas that are less prepared to handle the consequences and have fewer resources than many of their urban counterparts.^2^ In particular, the Appalachian region is among the most vulnerable regions due to demographics and lack of resources. Additionally, the per capita income of the Appalachian region is 20% lower than the average household income in the U.S., and those living in Central Appalachia are nearly 40% lower than the U.S. average.^3^ This confluence of factors suggests that people living in the Appalachian region are particularly vulnerable to the impacts of business closures due to the COVID-19 pandemic.

In addition to potential economic impacts faced by those who are unable to continue working from home, frontlines workers bear significant physical and mental health risks by caring for those with COVID-19 and continuing to have contact with the public.^4^ A large U.S. study of healthcare workers reported that 93% were experiencing stress and 86% anxiety during COVID-19.^5^ An emerging literature suggests that symptoms of stress, depressive disorder, and anxiety disorder have increased considerably in the U.S. since the beginning of the pandemic.^6^,^7^ Recent representative panel surveys among adults across the U.S. in June of 2020 found that 41% of respondents reported at least one adverse mental or behavioral health condition in response to the pandemic, including anxiety disorder, depressive disorder, and symptoms of trauma.^8^ Again, Appalachians are at disproportionate risk compared to other Americans, with limited resources and an inferior healthcare infrastructure.

The purpose of this research was to examine the mental health and financial and healthcare issues faced by Appalachian residents during the COVID-19–related shutdown in Spring of 2020, and to determine if there were differences in these outcomes by those who continued working outside the home or switched to working remotely during the shutdown. The primary hypothesis tested was that those who continued working on the frontlines during the shutdown, would have higher levels of anxiety, fear, and depression than those who did not work on the frontlines.

## METHODS

A survey was developed as a collaboration between academic and community partners to explore a wide variety of issues related to COVID-19 and the resultant shutdown. For this substudy, questions investigated employment status, financial and healthcare issues, mental health, disruption of life events and thoughts and feelings due to COVID-19. Some questions were drawn from surveys available from the NIH National Public Health Emergency and Disaster Response Research (DR2) website.^9^ Questions were revised based on feedback from Appalachian community partners to ensure the appropriateness of the survey questions and response options for the Appalachian region. Single and multiple-choice responses were provided along with free-form narrative descriptions to enable personal experiences to be captured.

Study data were collected and managed using Research Electronic Data Capture (REDCap) electronic data capture tools hosted at the University of Kentucky.^10^,^11^ REDCap is a secure, web-based software platform designed to support data capture for research studies, providing (1) an intuitive interface for validated data capture; (2) audit trails for tracking data manipulation and export procedures; (3) automated export procedures for seamless data downloads to common statistical packages; and (4) procedures for data integration and interoperability with external sources.

The initial participant pool included friends and colleagues of the research team. Existing study populations were also invited to participate, providing a convenience sample, within the Appalachian region. An email directed recipients to study information, consent form and survey through a link to a page on the University of Kentucky’s College of Public Health webpage. Participants then shared the link with others, often through Facebook, resulting in snowball sampling. Survey access was provided through a REDCap link from May 8 to June 6, 2020. The University of Kentucky Office of Research Integrity approved the human subject research.

For this study, survey items relating to working status prior to and during the COVID-19 shutdown were used to define comparison groups. Participants were first classified based on working outside the home prior to COVID-19. Three working group categories were used: not employed outside the home prior to COVID-19, switched to working remotely, and continued working outside the home, i.e., essential frontline workers. Outcomes of interest for this study included financial and healthcare issues related to COVID-19. Survey responses were summarized by counts and percentages overall and by the three working categories; comparisons across the three working categories were made using chi-square tests of independence. Because we limited primary hypotheses, adjustments for multiple comparisons were not conducted. All analyses were performed using SAS v9.4 and all statistical tests used a significance level of 0.05.

## RESULTS

Our survey was completed by 751 individuals and 651 (87%) provided zip code allowing for county identification. This paper includes the 276 (42%) participants across nine states from counties designated as Appalachian by the Appalachian Regional Commission (ARC). A limited number of participants (n=17) lost their job due to COVID-19; these were excluded due to small sample size. The remaining participants (n=259) report female gender (80%), white race (95%), attended at least some college (90%), and approximately half (49%) were between the ages of 45 and 64 (Table 1).

Twenty-three percent (n=59) indicated not working outside the home prior to the COVID-19 shutdown. Of the remaining 77% who did work outside the home prior to COVID-19, 34% continued working outside the home and 43% did not continue working outside the home after the shutdown. Workers who continued working outside the home, reported the following essential and frontline occupations: health care (n=41), education (n=12), manufacturing (n=9), public service (n=5), construction (n=3), retail (3), and other (n=16).

Significant differences in age (p<0.01), gender (p<0.01), education (p<0.01), and health conditions (p<0.01) were observed across the three working group categories (Table 1). Compared to the other two categories, participants not working from home prior to COVID-19 tended to be older (42% 65 and older), without college education (22%), and reported at least one health concern (63%). Of note, a greater percentage of males continued working outside the home after shutdown.

Those who continued working outside the home reported more negative financial impacts (38%) than those who were not employed outside the home (20%), or those who switched to working remotely (27%, p=0.05). Of the financial impact categories, the leading issue identified by participants was lost or reduced income (24%) and this was reported by one-third of those who continued working outside the home (Table 2). While most participants did not experience loss of healthcare benefits, approximately half missed or postponed appointments; these rates tended to be higher for those not working outside the home (before or after shutdown).

Mental health impacts were similar across the groups, with two-thirds of the respondents reporting at least one mental health issue (66%). The leading issues experiences across the sample were isolation/loneliness (40%), depression (27%), and anxiety (52%). Although not significantly different, those who did not work outside the home prior to COVID-19, reported slightly higher isolation and loneliness (49%) than those who switched to working remotely (41%) (Table 2). Additionally, those continuing work outside the home were more likely to report feeling anxious (52%) and reported the highest levels of being overwhelmed by worries (24%), but this difference was not statistically significant. Those who did not work outside the home prior to COVID-19 reported significantly lower rates of feeling nervous (29%, p=0.02) and tense (24%, p=0.03) compared to those who switched to working remotely (50% and 44%) and those who continued working outside the home (51% and 42%). Those not working outside the home prior to COVID-19 and those who switched to working remotely also report higher rates of being very afraid or afraid and concerns around the seriousness of the effect of getting COVID-19 compared to those who continued working outside the home (p<0.01) and p<0.01), respectively (Table 2). Within our study, only 17% reported that their mental health had not changed or had not gotten worse due to COVID-19. Although the participants reported feeling nervous (45%), tense (39%), felt fearful (47%), and difficulty focusing on anything other than anxiety (25%), only 9% stated that they felt like they needed help for anxiety (Table 2).

## DISCUSSION

This study is the first to investigate reports of financial, health care, and mental health in Appalachians working during the shutdown due to the COVID-19 pandemic. Whereas the majority of research has focused exclusively on the mental health of healthcare workers,^12^ our data demonstrate that mental health is a considerable health outcome for all and experienced equally among those who switched to working remotely, and those who continued working as frontlines or essential workers during the COVID-19 shutdown. Rates of perceived stress and reports of depression were also considerable for all participants. Based on a recent Appalachian Regional Commission report,^13^ there is a higher prevalence of mental health disorders in the Appalachian region compared to the rest of the U.S. Thus, the shutdown due to the COVID-19 pandemic could potentially have far-reaching mental health impacts, particularly for those in the Appalachian region. Research on the impact of the pandemic on mental health has linked the COVID-19 pandemic with increases in stress and depressive symptoms as well as declines in well-being around the U.S. and the world.^14^,^15^ Future research on mental health related to the pandemic should also include understanding the barriers for seeking mental health assistance or care, particularly in Appalachia where mental health issues are often associated with stigma and cultural value of self-reliance.^16^

Nearly a quarter of our sample experienced a loss or reduced income during the shutdown. This was significantly greater among those who continued working outside the home. In a Pew Research Center survey conducted in April 2020, 43% of U.S. adults reported that that they or someone in their household has lost a job or taken a cut in pay due to the outbreak.^17^ Similar to our study, Saloner and colleagues found that nearly 30% of their cross-sectional nationally representative online survey of American adults, experienced employment reduction (job loss or reduced earnings).^18^ Reduction in income was higher in a similar online survey conducted in Spring 2020 of rural residents in Pakistan where 64% of the respondents reported a decrease in income due to COVID-19.^18^,^19^ Financial concerns have long contributed to poor mental health and most recently to serious psychological distress,^18^,^20^ thereby potentially exacerbating the long-term consequences on mental health in the Appalachian region. The majority of our Appalachian participants did not experience loss of healthcare benefits, but nearly half missed or postponed a healthcare appointment. This is concerning given the current health status of the Appalachian population. The Appalachian region has higher rates of mortality than the nation resulting from heart disease, cancer, chronic obstructive pulmonary disease (COPD), injury, stroke, diabetes, and suicide.^21^ Successful control of these chronic diseases requires collaboration with health professionals. A study of diabetic patients found that adherence to appointments was a strong predictor of diabetic metabolic control.^22^ Thus, our participants who missed appointments due to the shutdown may suffer negative health consequences.

These results are qualified by a few limitations. First, reports of stress and mental health were collected at a single time during the pandemic. Without assessments of stress and mental health in our cohort before the pandemic, we were unable to examine changes in stress and mental health in response to the pandemic. Instead, our study assessed anxiety-related thoughts and feelings due to COVID-19 and general perceptions of stress and feelings of depression. Future research should include longitudinal designs to examine changes in stress and mental health across working groups. Doing so will better capture the continuing uncertainty and changing nature of the pandemic and may produce more fine-grained distinctions in stress and mental health. Second, participants in our sample were predominately female and Caucasian, likely due to our initial participant invitations and the snowball recruitment methodology and had a higher-than-average income relative to the entire Appalachian region. Given this, the generalizability of the results to the rest of the region may be limited.

This study is the first to examine mental health, financial and healthcare issues experiences by Appalachian residents during the COVID-19 shutdown. Based on these results, we recommend effective interventions designed to enhance psychological resilience and improve mental health and well-being are made available to everyone who experienced COVID-19. Providing tailored mental health and well-being interventions could equip individuals with the skills needed to combat the natural response of fear, anxiety, and depression in the face of growing uncertainty.^23^ Personalized and culturally appropriate approaches are likely to be an important component to address complex mental health conditions. These interventions will be essential to support and optimize coping strategies to mitigate symptoms of stress and anxiety.

**SUMMARY BOX**

**What is already known about this topic?** Many U.S. businesses shutdown in Spring 2020 to slow the spread of COVID-19. Research has focused on the impact of COVID-19 on frontlines workers, predominantly those in health care professions.

**What is added by this report?** This report identifies the financial and healthcare issues and mental health impacts experienced by Appalachians during the COVID-19 shutdown. Our survey data of 276 Appalachian participants indicated that nearly 25% had lost their job or had a reduction in income due to the shutdown. Approximately 50% missed or postponed health care appointments. Our data indicate that approximately 50% experienced anxiety regardless of working status: not working outside the home prior to the shutdown, continued working remotely, or those who continued working outside the home, i.e., essential workers.

**What are the implications for future research?** This work indicates that future research will be needed to explore health outcomes due to missed or postponed health care visits. Further research is also needed to identify the mental health and well-being impacts of those not working (i.e., retired), or those working remotely who are not on the frontlines of the pandemic. This work provides evidence to support the need for interventions to improve mental health and well-being for everyone who experienced COVID-19.

## Figures and Tables

**Table 1:** Characteristics of survey participants by work categories before and during the COVID-19 shutdown

		Working Status Prior to and During the COVID-19 Shutdown			
	Study Sample (%)	Did not work outside home prior to shutdown % (n)	Switched to working remotely during shutdown % (n)	Continued working outside home during shutdown % (n)	p-value
	N = 259	N = 59	N = 111	N = 89	
**Age**					**<0.001**
18–34	11	3 (2/59)	12 (14/111)	15 (13/89)	
35–44	26	9 (5/59)	30 (33/111)	31 (28/89)	
45–64	49	46 (27/59)	49 (54/111)	53 (47/89)	
65 and older	14	42 (25/59)	9 (10/111)	1 (1/89)	
**Gender, Female**	80	85 (49/59)	88 (97/111)	67 (58/89)	**<0.001**
**Race, Caucasian**	95	97 (56/58)	93 (102/110)	95 (84/88)	0.96
**Health Condition Present**	**46**	**63 (37/59)**	**44 (49/111)**	**37 (33/89)**	**0.008**
Moderate/Severe Asthma	7	10 (6/59)	6 (7/111)	6 (5/89)	
Chronic Lung Disease	2	2 (1/59)	0 (0/111)	3 (3/89)	
Diabetes	11	27 (16/59)	6 (7/111)	7 (6/89)	
Obesity	32	41 (24/59)	31 (34/111)	28 (25/89)	
Cardiovascular Disease	8	17 (10/59)	6 (7/111)	6 (5/89)	
Immunocompromised	8	15 (9/59)	7 (8/111)	3 (3/89)	
Chronic Kidney Disease	1	2 (1/59)	1 (1/111)	0 (0/89)	
**Income**					0.24
<$50,000	22	29 (14/48)	19 (19/99)	20 (17/84)	
$50,000 – $99,999	37	42 (20/48)	32 (32/99)	39 (33/84)	
≥ $100,000	41	29 (14/48)	49 (48/99)	41 (34/84)	

Note: P-values based on chi-square test of independence, comparing the three working categories: Did not work outside home prior to COVID-19, switched to working remotely, and continued working outside home.

**Table 2:** Financial, Health Care, and Mental Health Impacts of COVID-19 by work categories

		Working Status Prior to and During the COVID-19 Shutdown			
	Study Sample (%)	Did not work outside home prior to shutdown % (n)	Switched to working remotely during shutdown % (n)	Continued working outside home during shutdown % (n)	p-value
	N=259	N = 59	N = 111	N = 89	
**Financial**	**29**	**20 (12/59)**	**27 (30/111)**	**38 (34/89)**	**0.05**
Not Enough Money for Housing	3	0 (0/59)	2 (2/111)	6 (5/89)	
Not Enough Money for Car Payment	2	0 (0/59)	3 (3/111)	3 (3/89)	
Not Enough Money for Food	3	3 (2/59)	4 (4/111)	3 (3/89)	
Not Enough Money for Utilities	3	2 (1/59)	2 (2/111)	6 (5/89)	
Had to Use Credit Card to Cover Basic Needs	10	8 (5/59)	9 (10/111)	11 (10/89)	
Lost or Reduced Income	24	14 (8/59)	22 (24/111)	33 (29/89)	
**Health Care**	**60**	**63 (37/59)**	**64 (71/111)**	**52 (46/89)**	**0.18**
Lost healthcare benefits	1	2 (1/59)	0 (0/111)	1 (1/89)	
Missed/Postponed healthcare appointments	47	51 (30/59)	50 (55/111)	40 (36/89)	
Unable to be with family in health emergency	29	32 (19/59)	32 (35/111)	22 (20/89)	
**Mental Health**	**66**	**61 (36/59)**	**69 (76/111)**	**65 (58/89)**	**0.62**
Isolation/Loneliness	40	49 (29/59)	41 (45/111)	34 (30/89)	
Depression	27	27 (16/59)	25 (28/111)	30 (27/89)	
Hopelessness	8	7 (4/59)	8 (9/111)	10 (9/89)	
Anxiety	52	49 (29/59)	54 (60/111)	52 (46/89)	
**Thoughts and Feelings Due to COVID-19**					
Felt nervous	45	29 (17/59)	50 (55/111)	51 (45/89)	**0.016**
Felt tense	39	24 (14/59)	44 (49/111)	42 (37/89)	**0.026**
Worries overwhelmed me	19	17 (10/59)	17 (19/111)	24 (21/89)	0.45
Felt fearful	47	37 (22/59)	53 (59/111)	46 (41/89)	0.14
Felt uneasy	60	59 (35/59)	63 (70/111)	57 (51/89)	0.70
Hard to focus on anything other than anxiety	25	15 (9/59)	27 (30/111)	30 (27/89)	0.11
Felt like I needed help for my anxiety	9	8 (5/59)	8 (9/111)	10 (9/89)	0.88
No change/Has not gotten worse	17	20 (12/59)	14 (16/111)	19 (17/89)	0.54
**COVID-19 Related Concerns**					
Very/Afraid of getting it	32	42 (25/59)	36 (40/111)	20 (18/89)	**0.009**
Very/Serious if I got it	57	78 (39/50)	55 (55/100)	47 (37/79)	**0.002**
Very/Afraid household will get it	45	52 (27/52)	49 (51/104)	37 (31/84)	0.14

Note: P-values based on chi-square test of independence, comparing the three working categories: Did not work outside home prior to COVID-19, switched to working remotely, and continued working outside home.
